# Impact, use, and implications of artificial intelligence in public health decision making by elected officials: a scoping review

**DOI:** 10.3389/fpubh.2026.1745684

**Published:** 2026-03-03

**Authors:** Elizabeth A. Campbell, Hannah Goodtree, Sarah Gillani, Oluremilekun Oyefolu, Alison Kelly, Caitlin Rivers, Crystal Watson

**Affiliations:** 1 Johns Hopkins University Center for Health Security, Baltimore, MD, United States; 2 Johns Hopkins Center for Outbreak Response Innovation, Baltimore, MD, United States

**Keywords:** AI, COVID - 19, elected officials, government, public health decision making, public health emergencies (PHE)

## Abstract

**Introduction:**

Artificial intelligence (AI) offers considerable promise for strengthening governmental public health decision making by supporting rapid, comprehensive analysis of complex data. Although AI applications have been widely examined in clinical and academic settings, their use in public health agencies and policymaking remains less well understood.

**Methods:**

This scoping review assessed how AI has been applied to support decision making by public health professionals and elected officials in both routine and crisis contexts. Using PRISMA-ScR guidelines, we searched PubMed, OAISTER, and Web of Science for literature published between 2014 and 2024. From 13,239 records identified, seven studies met final inclusion criteria.

**Results:**

The identified evidence base is primarily descriptive and exploratory, with limited empirical evaluation of outcomes or effectiveness. All included studies described AI use during the COVID-19 pandemic, focusing on vaccination decision support, contact tracing, quarantine enforcement, and/or movement restrictions, which limits generalizability to other public health contexts and decision-making scenarios. Findings highlight a small but emerging evidence base, with most applications developed in response to emergencies rather than embedded in routine practice.

**Discussion:**

Future opportunities for AI include advancing surveillance, communication, and resource allocation. However, critical challenges remain regarding governance, equity, and implementation. Further research is needed to evaluate AI interventions in diverse contexts and establish sustainable pathways for adoption by governmental public health agencies.

## Introduction

### AI applications in government

Public health decisions about intervention planning, resource allocation, emergency response, and long-term policy formulation are usually made by governmental public health experts, be they in local/tribal, state/territorial or federal positions. However, in some cases, public health-related decisions are made not at the expert level, but by an elected official such as a mayor or governor. Regardless of decision-maker or context, high-quality information and analytical capabilities for identifying, accessing, and processing data to support these decisions is necessary (1, 2). Artificial intelligence (AI) may be a valuable tool that public health officials and elected leaders can incorporate as one input to decisions. AI and machine learning can facilitate access to a broader set of data, more quickly, and may be helpful for an expert or leader to see a more complete picture of the available data and to understand tradeoffs inherent to a decision.

We define AI as a branch of computer science that focuses on building tools that represent characteristics of the human mind and have the capacity to complete intellectual tasks that humans can perform (3, 4). Machine learning (ML), natural language processing, and image and signal processing are all AI subfields that may be used individually or within a single application (5). Broadly, AI has shown potential to help public health experts and elected officials navigate complex decision-making environments and promote technological growth, foster sustainability, and improve quality of life (6, 7). Digital health interventions, such as telemedicine, wearable devices, electronic medical records, and mHealth, have already led to significant advances in public health research and practice, including affordable and accessible health education modalities and chronic disease self-management (8, 9). Digital health technologies generate massive amounts of data that AI-driven analysis may be used to better understand real-time symptom tracking, health services demand, and intervention efficacy (10, 11). AI may aid in analyzing large datasets for disease surveillance and prediction, contact tracing, and other activities that inform government programs, policies, and outbreak responses (12). Although numerous potential applications have been proposed in the literature, the use of AI in public health decision-making remains limited. While limited, AI has been used in a government context to predict child maltreatment (13), hospital patient loads, and forest fires (14). Potential further applications of AI in government and public services include using predictive analytics to plan for resource allocation (e.g. emergency responses to extreme weather events), improving public safety, and forecasting economic and market trends (15, 16). However, it is important to note that empirical documentation of actual implementation by governmental decision makers is sparse.

### Potential uses for AI in crisis public health decision-making, particularly for elected officials

As was demonstrated clearly during the COVID-19 pandemic, public health crises can precipitate a shift of responsibility for some high-consequence public health decisions to elected leaders such as mayors and governors. Sometimes this shift in decision-making authority occurs because there is a complex set of non-public health factors (e.g. social, economic, political) that must be considered carefully. Sometimes this shift happens because of the intense public scrutiny that comes with emergency response- elected leaders may want to have more control over decisions when they will be held personally responsible for the outcomes (17). In these emergency situations, public health experts often provide support for decision-making as part of an advisory body. However, especially in urgent and complex emergencies, there may be a need for additional decision-support resources and tools for elected leaders, who may not have public health training or previous experience with response to a public health emergency. AI has significant potential as a tool for elected officials in making these public health decisions, especially when paired with a diverse set of expert advisors.

### Scoping review objectives

In this work, we present findings from a scoping review to assess current literature on AI applications for public health professionals and elected officials in both routine public health decisions and crisis decisions during an emergency response. Our focus is on elected officials, which we define as individuals in politically accountable positions with direct authority over policy decisions, while recognizing that these officials often work alongside public health professionals in the decision-making process. For this review, we include studies where AI tools were used to inform decisions ultimately made by elected officials or governing bodies. This review helps to contextualize current literature on how AI may be used to support public health decision making efforts, provides recommendations, and identifies areas for future research. The literature review aims to address the following research questions:

1. How has AI been used to support decision making in routine public health practice and in emergency response?
2. How can governmental agencies and decision makers use AI in public health efforts in the future?

## Materials and methods

The literature search process, review strategies, and inclusion and exclusion criteria were developed in accordance with the Preferred Reporting Items for Systematic Reviews and Meta-Analysis Extension for Scoping Reviews guidelines (PRISMA-ScR) (18). The databases that were used in this search were PubMed, OAISTER, and Web of Science. Literature published from January 1, 2014, to December 31, 2024, were included to capture research and governmental response efforts spanning the 2014–2016 West Africa Ebola epidemic and the COVID-19 pandemic, two of the most significant infectious disease public health challenges of the 21st century.

Articles identified in the initial database scan were retained for the review based on the following inclusion and exclusion criteria:

*Inclusion Criteria*:

- Peer-reviewed full publications.
- Governmental and non-Governmental reports and white papers.
- Empirical studies.
- Includes a discussion on utilizing artificial intelligence to aid in elected officials' or public health officials' decision-making processes in a public health context.
- May include studies utilizing quantitative, qualitative, or mixed methods approaches to data analysis.

*Exclusion Criteria*:

- Conference proceedings/abstracts, posters, and book chapters.
- Review articles.
- Not English language.
- Published before 2014.

### Operational definitions: decision-making authority

For this review, we defined “governmental public health decision-making” broadly to encompass decisions made by individuals with statutory or delegated authority to implement public health interventions, allocate resources for public health programs, or establish public health policies. This includes:

- Elected officials: mayors, governors, county executives, city council members, legislators, and other individuals in elected positions at local, state/provincial, and national levels who exercise decision-making authority over public health matters.
- Appointed public health professionals in governing bodies: health directors, commissioners, chief medical officers, and other appointed officials with delegated authority to make or recommend public health decisions.

Using the MeSH database (19) as a starting point, we developed a keyword list (Table 1) to represent key concepts for the scoping review, namely “Artificial Intelligence,” “Government,” and “Public Health” in the PubMed Database. Given the diversity of government contexts that AI may be deployed in, we considered keywords related to local, state, and federal government along with government agencies.

**Table 1:** AI applications in decision making for public health officials scoping review keywords.

**Concept**	**Search terms**	
Artificial intelligence	“Artificial Intelligence” OR “Intelligence, Artificial” OR “Computer Reasoning” OR “Reasoning, Computer” OR “AI (Artificial Intelligence)” OR “Machine Intelligence” OR “Intelligence, Machine” OR “Computational Intelligence” OR “Intelligence, Computational” OR “Computer Vision Systems” OR “Computer Vision System” OR “System, Computer Vision” OR “Systems, Computer Vision” OR “Vision System, Computer” OR “Knowledge Acquisition (Computer)” OR “Acquisition, Knowledge (Computer)” OR “Knowledge Representation (Computer)” OR “Knowledge Representations (Computer)” OR “Representation, Knowledge (Computer)” OR ”Machine Learning” OR “Deep Learning” OR “Supervised Machine Learning” OR “Support Vector Machine” OR “Unsupervised Machine Learning” OR “Computer Heuristics” OR “Expert Systems” OR “Fuzzy Logic” OR “Natural Language Processing” OR “Neural Networks, Computer” OR “Robotics” OR ”Sentiment Analysis” OR “Date Science”	AND
Government	“Federal Government” OR “Government, Federal” OR “National Government” OR “Agencies, Government” OR “Agency, Government” OR “Government Agency” OR “Government, Municipal” OR “State Government” OR “Government, State” OR “State Governments” OR “Provincial Government” OR “Government, Provincial” OR “Government, Local” OR “Local Government” OR “City Government” OR “Government, City” OR “County Government” OR “Government, County” OR “Metropolitan Government” OR “Government, Metropolitan” OR “Municipal Government” OR “Official” OR “Decision Maker” OR “Decision Making” OR “Government”	AND
Public health	“Public Health” OR “Health, Public” OR “Community Health” OR “Health, Community” OR “Environment, Preventive Medicine and Public Health” OR “Outbreak” OR “Pandemic” OR “Epidemic” OR “Outbreak Response” OR “Emergency”	

The OAISTER database required that searches be no longer than 30 words, so we used the following adjusted query to meet these guidelines:

(“Artificial Intelligence” OR “Computer Reasoning” OR “AI (Artificial Intelligence)” OR “AI” OR “Machine Intelligence” OR “Machine Learning” OR “Deep Learning” OR “Supervised Machine Learning” OR “Unsupervised Machine Learning” OR “Data Science”) **AND** (“Federal Government” OR “National Government” OR “Government Agency” OR “Municipal Government” OR “State Government” OR “State Governments” OR “Provincial Government” OR “Local Government” OR “City Government” OR “Government, County” OR “Metropolitan Government” OR “Official” OR “Decision Maker” OR “Decision Making” OR “Government”) **AND** (“Public Health” OR “Community Health” OR “Environment, Preventive Medicine and Public Health” OR “Outbreak” OR “Pandemic” OR “Epidemic” OR “Outbreak Response” OR “Emergency”).

All literature identified through the searches underwent title and abstract screening, conducted by 2 separate reviewers, to determine whether each met the criteria for full-text review. A full-text review of applicable articles was conducted in Covidence (20) by 2 separate reviewers to assess if they merited inclusion in the final analysis. Data from the articles that merited inclusion was extracted into a form by 2 separate reviewers. At each stage of the analysis process, any disagreements between the reviewers were resolved by discussion and consensus among the 2 initial reviewers or, if consensus could not be reached, via adjudication by a third reviewer. The data extracted included the study period, country or location of study, AI intervention, study design, governmental decision making that the AI intervention supported, and limitations. Because scoping reviews aim to map the breadth of available evidence rather than assess study quality or synthesize effectiveness, we did not conduct formal quality appraisal of included studies.

## Results

**Figure 1:**
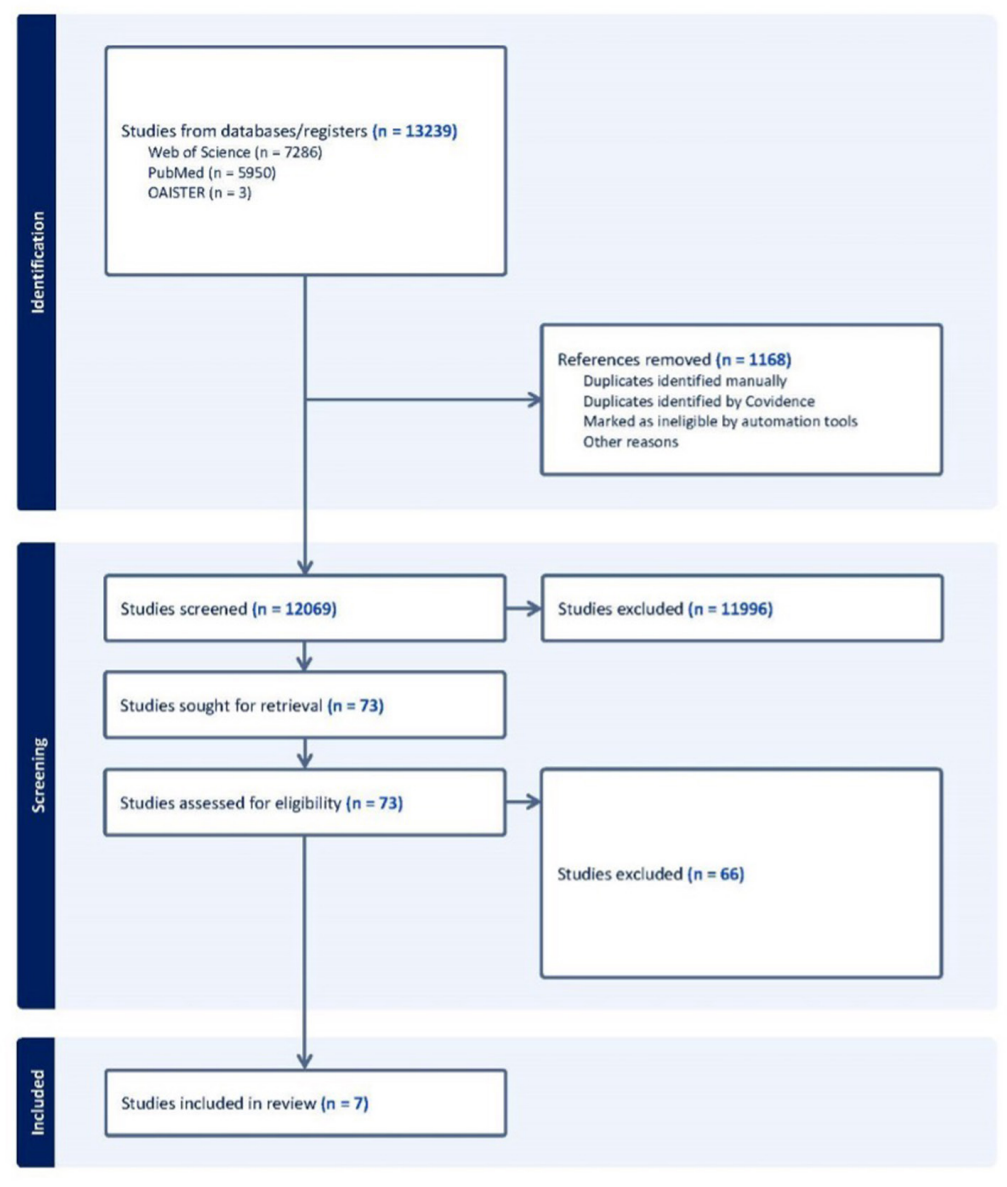
AI Scoping review (public health decision making).

Initial database scans returned 13,239 studies, of which *n* = 1,168 duplicates were removed leaving 12,069 studies screened. After abstract screening, *n* = 11,996 articles were excluded and *n* = 73 underwent a full text screening. Of the 73 studies that underwent full text screening, *n* = 66 were excluded and *n* = 7 met the criteria for inclusion in the study. Figure 1 presents the article identification, screening, and inclusion processes for this review.

Table 2 presents the data extraction table developed for the seven articles that were included in the review.

**Table 2:** The presents data extracted from studies (*n* = 7) that were included in the scoping review.

**Study**	**Extracted data**
Dong et al. (27)	*Study Design:* Descriptive Study *Setting:* China *Public Health Problem: COVID-19* *Study population:* Chinese citizens and Government *Data Sources:* Literature review that included data sources from epidemic data, medical, government, public, and media response data, traffic flow data, personal information, travel data, public security data, mobile phone data, and clinical data. *AI/ML Intervention:* China National Center for Bioinformation developed big data/AI technology to develop diagnosis and treatment protocol for COVID-19; app-based data acquisition for confirmed COVID-19 cases for analysis; COVID-19 virus database *Decision Making Effort:* Decisions on closing water, land, and air transportation and mobilizing medical workers/public health professionals to support the city; contact tracing and tracking sick individuals and drawing epidemic maps *Limitations:* Limited detail on specific governmental decisions that AI supported and specifics of design for the AI interventions, application of big data and AI technologies in sudden public health events lack validation of repeatability and universality
Sharin et al. (23)	*Study Design:* Descriptive study *Setting:* Malaysia *Public Health Problem:* COVID-19 *Study population:* Malaysian citizens *Data Sources:* Malaysian Ministry of Health data, including cases by state, country wide, and deaths country-wide. *AI/ML Intervention:* Network analysis and support vector machine to visualize connectivity and predict pandemic risk (number of COVID-19 cases) based on confirmed COVID-19 cases and deaths *Decision Making Effort:* Preventive measures in highly populated states/conditional movement control orders *Limitations:* Limited specifics on how the AI/ML intervention was incorporated into government policy/decision making
Basso et al. (21)	*Study Design:* Observational/quasi experimental *Setting:* Chile *Public Health Problem:* COVID-19 pandemic management *Study population:* Chilean citizens *Data Sources:* Mobility patterns collected from cellphone data, demographic data from the Chilean census, serology data, and COVID-19 repository and epidemiological data from the Chilean Ministry of Health. *AI/ML Intervention:* Developed a Decision Support Dashboard that incorporated machine-learning models *Decision Making Effort:* Dashboards and models enabled decision-makers/users to act regarding contagion prevention, management of hospital resources, and vaccination roll-out. *Limitations:* Intervention was enacted during a global pandemic and had to navigate extreme stress while building trust, coordination, and cooperation
Renukappa et al. (24)	*Study Design:* Literature review and semi structured interviews *Setting:* India *Public Health Problem:* COVID-19 *Study population:* Indian citizens *Data Sources:* Interviews of 20 smart city experts in India *AI/ML Intervention:* Developed a mobile app to track symptoms and alerts municipal doctors, access emergency services, provide local updates and government announcements, and assist with contact tracing. Developed AI predictive analytics using heat maps and movement monitoring, and periodic health status updates for suspected cases *Decision Making Effort:* Government communication to the public and managing symptoms and contact tracing/connecting individuals with government resources *Limitations:* Limited details on the specifics of predictive analytics developed by governmental organizations and the decision making those findings guided
Alfattani et al. (26)	*Study Design:* Descriptive Study *Setting:* Saudi Arabia *Public Health Problem:* COVID-19 *Study population:* Citizens of Saudi Arabia *Data Sources:* Data entered into app by users, movement data, vaccination and COVID-19 case data from Saudi Ministry of Health *AI/ML Intervention:* Tawakkalna App developed to facilitate travel permits during Hajj. The app is integrated across government systems and fed by real-time inputs and GPS data. The app was used to monitor movements during quarantine, to give notifications to users when they have been in an area of positive cases, and to prove vaccination status. Developed Tatamman: electronic bracelets to track positive cases *Decision Making Effort:* The app and e-bracelets helped policymakers make informed decisions for preventative measures and strategies. *Limitations:* Did not expand on the precautionary measures, policies, and strategies that AI interventions informed
Wang et al. (25)	*Study Design:* Descriptive study (qualitative methods) *Setting:* China *Public Health Problem:* COVID-19 *Study population:* Chinese citizens *Data Sources:* Chinese government documents, WHO and local public health officials' comments, and reputable media reports. *AI/ML Intervention:* The government designed a platform to identify confirmed cases and close contacts and to monitor their body temperatures. Technologies were used to strengthen information processing, efficient health management and launching health code. *Decision Making Effort:* Contact tracing, developing guidelines, making decisions regarding return to work, information dissemination *Limitations:* Limited discussion of what specific policies developed based on AI were
Hyun Lim et al. (22)	*Study Design:* Descriptive study *Setting:* Canada *Public Health Problem:* Vaccine guidance development during COVID-19 (and synthesizing massive amounts of COVID-19 research that was generated to inform public health policy) *Study population:* Public Health Agency of Canada (PHAC), COVID-19 vaccine-related literature *Data Sources:* Literature review *AI/ML Intervention:* Evidence extraction team for research analysis: AI screening to develop a bibliographic repository to support decision making by the National Advisory Committee on Immunization; trends in vaccine research were analyzed over time *Decision Making Effort:* Informed situational awareness and developing timely vaccine guidance on vaccination and booster doses, myocarditis and timing of vaccination in individuals who were previously infected, heterologous primary series, and off-label additional doses for immunocompromised populations *Limitations:* Single reviewer at each stage of the literature curation process, efficiency was prioritized which may limit the use of the repository for other purposes

The findings from this review highlight the themes that emerge from the field of AI and public health decision making. The seven extracted studies all focus on interventions in the COVID-19 pandemic, which represents a turning point in data-driven decision making by governments for public health emergencies. Two articles utilize the AI/ML data and outputs for vaccination decision making, including vaccine roll out, developing vaccine guidance, timing of doses, and use of off-label doses for specific populations (21, 22). The other five articles all discuss how AI/ML interventions were used to help determine contact tracing, quarantine orders, lockdown timing, and movement control orders.

An important finding from this review is the need to distinguish between AI as an analytical tool and AI as a decision support system - a distinction that has implications for understanding AI's actual role in governmental decision-making. Analytical tools use AI to process and analyze data, generating insights, visualizations, or summaries that decision-makers subsequently interpret and incorporate into their deliberations, whereas decision support systems provide specific recommendations or action items directly linked to particular decisions. Importantly, the included studies varied in how explicitly they linked AI outputs to policy decisions. In some cases (*n* = 3) (23–25), AI served primarily as an analytic tool generating information for decision-makers, with limited documentation of how outputs influenced actual policy choices. In other cases (*n* = 2) (22, 26), studies reported more direct connections between AI-generated recommendations and implemented policies. Still others (*n* = 2) (21, 27) described both an analytical innovation as well as how analysis and implementation informed policy making. This ambiguity in the literature limits our ability to assess AI's actual influence on governmental decision-making.

Data sources found in the reviews varied. Some studies exclusively used literature review findings and did not collect, analyze, or utilize original data sources (20, 26). Other studies used government or ministry of health data (21, 23, 25, 26), while others used individual data sources like data collected from app users or interviews (24, 26).

Limitations in these studies centered on limited specifics or details on how policies or interventions were developed using AI/ML as decision making tools. In Lim 2024, the authors noted that their limitations were only using a single reviewer during their literature review (22), while Basso 2023 noted that their study was limited by the COVID-19 pandemic which strained cooperation and trust between different entities that participated in their research (21).

It is critical to note that the limited number of included studies (*n* = 7) reflects a nascent and fragmented published literature rather than necessarily indicating an absence of real-world AI use by elected officials. Governmental AI implementation may be occurring but not systematically documented in peer-reviewed or gray literature accessible through our search strategy.

## Discussion

The preceding study is a scoping review of literature published between 2014 and 2024 to examine the use of AI in governmental public health decision making. Despite growing interest in AI applications across the health sector, relatively few studies directly explored how AI has been operationalized to support political or policy decisions during public health emergencies. This suggests that while the development of AI tools is expanding, their deployment and scale-up within governmental settings remain in its nascent stages.

Much of the existing literature on AI interventions for public health is concentrated in clinical or academic environments (28). A substantial body of research has focused on forecasting disease transmission, optimizing resource allocation, and improving diagnostics using machine learning and other AI techniques. However, these interventions were largely developed and evaluated within academic institutions or healthcare systems, with limited attention given to their integration into public health agencies or policy frameworks. Approximately 39% of the studies reviewed focused on the use of AI in clinical settings.

Moreover, many interventions were developed in response to the COVID-19 pandemic. Of the studies screened, approximately 29% either mentioned COVID-19 or focused specifically on it, underscoring the pandemic's role as a major catalyst for AI research in public health. This trend may reflect the urgent need for innovative, rapid-response tools during crises where traditional systems are strained by limited resources and the fast pace of emerging threats. However, despite the proliferation of COVID-19-related AI tools, few studies examined how these tools informed real-time governmental decision making.

For example, several studies demonstrated the use of AI to triage patients admitted to emergency departments or diagnose COVID-19 via imaging technologies such as CT scans (28–30). Yet these models were rarely linked to decision-making processes within public health agencies. Many forecasting tools were reviewed retrospectively in academic settings, rather than used operationally. Other studies proposed promising applications—such as predictive analytics for identifying high-risk populations (31) or anticipating disease spread—but stopped short of describing adoption by public agencies or evidence of influence on high-level decision making (31, 32).

The use of AI in clinical settings is far better documented than its use in governmental public health contexts (28, 32). Although academic literature frequently discusses the potential of AI for public health applications, few studies validate these tools in real-world settings (31). There is a pressing need to document not only successful implementations but also the challenges and limitations of integrating AI into public health decision making. Such documentation is critical for institutional learning as public health systems increasingly explore the use of AI-based interventions.

Overall, few studies provided insight into how AI-generated outputs were translated into actionable policies or decisions by governmental bodies. The disconnect between the development of AI models, whether and how they are used operationally, highlights a significant gap in the literature. Furthermore, there is limited understanding of how decision-makers interpret and apply AI-generated insights, or how such tools are integrated into existing public health workflows, especially during emergencies. It is possible that AI tools are being utilized in government contexts, but these use cases remain undocumented in the peer-reviewed literature. This represents a critical gap in knowledge generation and underscores the need for systematic reporting on the use of AI in public health decision-making.

### Challenges and opportunities for AI implementation in public health

Based on the limited evidence identified in this review, several critical questions remain unanswered. None of the reviewed studies included comparative analyses or outcome evaluations, preventing conclusions about whether AI-supported governmental decisions led to better public health outcomes, improved decision quality (accuracy, comprehensiveness, timeliness), or offered advantages over existing analytical methods and expert judgment. The studies describe AI tool development and deployment but do not assess impact on population health metrics, epidemic control, or the actual influence of AI outputs on policy decisions. Additionally, we lack evidence on adoption patterns. This includes rates of sustained operational use, factors influencing governmental officials' choices to utilize AI tools, and the pathway from AI-generated insights to enacted policies.

All seven included studies focused exclusively on COVID-19 pandemic response, preventing assessment of whether these applications would transfer effectively to routine public health decision-making, other types of emergencies, or different governmental contexts. The unique characteristics of the COVID-19 pandemic (unprecedented data availability, intense public attention, rapid technological mobilization) may limit generalizability. Furthermore, while equity concerns are discussed theoretically in the broader literature, no studies empirically assessed whether AI use affected health equity, exacerbated disparities, or distributed benefits and burdens equitably across populations. These substantial gaps highlight the nascent state of evidence in this field. While AI tools have been developed and deployed during the COVID-19 pandemic, rigorous evaluation of their role, effectiveness, and impact in governmental public health decision-making has not yet been conducted.

The challenges and opportunities discussed below synthesize insights from the included studies alongside broader literature on AI governance and public health decision-making. Many of these considerations extend beyond what was explicitly documented in the seven included studies and represent areas warranting attention in future research and practice. The identified gaps in the literature are a catalyst for distinguishing future areas for growth. Artificial intelligence presents a strategic opportunity to enhance public health decision-making and particularly public health crisis response practice. First, advanced surveillance systems can analyze diverse data sources simultaneously to detect emerging disease patterns before they become apparent through traditional monitoring approaches (33, 34). Second, customized health communication becomes achievable as artificial intelligence generates messages precisely tailored to audience characteristics, thereby significantly improving information accessibility across diverse populations and cultural contexts (35). Third, workflow optimization through intelligent automation could liberate public health professionals from administrative burdens, enabling greater focus on community engagement and strategic initiatives (36). Fourth, data-driven decision support becomes more robust as AI models synthesize complex epidemiological, environmental, and social data to forecast trends and evaluate interventions (37, 38). These capabilities may provide public health leaders with unprecedented tools to strengthen early warning systems, communication effectiveness, operational efficiency, and evidence-based practice, which are all critical components for addressing complex population health challenges in resource-constrained environments.

Despite opportunities created through AI to transform public health, the perceived benefits are accompanied by substantial challenges that demand immediate attention that future work may investigate. The rapid evolution of artificial intelligence has outpaced regulatory frameworks, creating a governance gap that leaves both individuals and institutions vulnerable (36, 39). Traditional de-identification protocols are increasingly insufficient against sophisticated re-identification algorithms, threatening the confidentiality of sensitive health information (39). Similarly, artificial intelligence systems with built-in prejudices threaten to exacerbate health disparities particularly for underrepresented communities and could potentially lead to distorted disease forecasting and inequitable resource allocation (36, 37). Operational challenges such as lack of integration into public health systems, workforce training gaps, and inadequate infrastructure, further complicate the effective implementation of artificial intelligence in public health (34).

### Strengths and limitations

In this review, there were several strengths and limitations. Our findings are strengthened by the comprehensive scope and size of this review. The literature review included 13,239 papers at onset and each phase of the review had at least 2 reviewers. Next, our review investigated a thorough and well-defined research question that has not yet been explored in the literature. However, although the review screened over 13,000 papers, only 7 were included in the final extraction process of the review. The included studies only discussed AI/ML interventions in the context of COVID-19, so there is little diversity, and most studies were descriptive studies. The review process also removed literature reviews and conference proceedings which might have contained additional examples. This also means that there has been minimal highly rigorous evaluation of AI intervention in public health decision making. Another important limitation is the primarily descriptive nature of included studies, with limited evaluative rigor or empirical assessment of outcomes. Most studies documented AI use or described AI outputs but did not rigorously evaluate whether AI improved decision quality, timeliness, or public health outcomes. This constrains our ability to draw conclusions about AI's actual impact on governmental decision-making effectiveness.

Furthermore, methodological constraints may have introduced bias and limited the scope of our findings. First, our restriction to English-language publications may have resulted in geographic and cultural bias. Countries with substantial AI investment and governmental AI applications that publish primarily in languages other than English may have been underrepresented in our review. This language restriction may have caused us to miss important implementations and lessons learned from diverse governmental contexts.

Although we included Governmental and non-Governmental reports and white papers in our search criteria, we may have excluded important gray literature including conference proceedings. Conference proceedings, in particular, often report early-stage implementations and pilot studies that may represent important real-world applications. The exclusion of these sources, while necessary for maintaining study quality standards, may mean our review captures primarily academic research rather than operational practice. Future work should endeavor to improve on these limitations and examine future AI/ML interventions in more expansive public health decision making processes and contexts.

Finally, the challenges identified in our discussion of AI implementation drew from both the primary studies in our review and the broader adjacent literature. Given that only seven studies met our inclusion criteria and all focused narrowly on COVID-19, supplementing with relevant literature from AI ethics, public health informatics, and governmental technology implementation was necessary to provide comprehensive context. However, this means some challenges discussed may not have been explicitly identified in studies specifically examining AI use by governmental decision-makers. We have attempted to clearly distinguish between findings from our primary review and insights from adjacent literature throughout the Discussion.

The small number of included studies (*n* = 7), their exclusive focus on a single public health crisis (COVID-19), and their predominantly descriptive rather than evaluative nature severely limits our ability to draw general conclusions about AI's role in governmental public health decision-making. The current evidence base describes AI applications but does not evaluate their effectiveness, impact, or appropriate role in public health governance.

## Conclusion

This scoping review mapped the limited published evidence on AI use for decision-making by elected officials during public health crises and highlights the limited integration of AI into public health decision making at the governmental level to date, compared to significant advances in AI applications in healthcare. Much of the identified extant literature focused on AI applications in clinical and academic settings. Studies that met our criteria for inclusion focused on operationalizing AI-supported tools for elected officials during the COVID-19 pandemic. However, an identified gap in the literature is a focus on how AI-supported applications and insights have been and could be incorporated into public health practice, policies, and decision making more broadly. Expanding AI usage represents a significant opportunity to modernize and expand governmental public health response, yet doing so requires careful evaluation, documentation, transparency, and safeguards.

Future research may focus on identifying new applications of AI for public health practice and decision-making, ensuring that AI's benefits are equitably distributed across the populations that public health serves, and that data privacy and confidentiality are upheld when AI is applied to de-identified and aggregate data. Governmental partners and regulatory bodies should be involved early in AI tool development to ensure that results are useful and meet operational challenges by closing the gaps between technological innovation and governmental adoption, AI may significantly enhance public health decision-making for future emergencies and public health needs.

## Data Availability

The original contributions presented in the study are included in the article/supplementary material, further inquiries can be directed to the corresponding author.
